# Complete Prolapsus and Separation of the Vagina

**Published:** 1842-08-06

**Authors:** 


*Complete Prolapsus and Separation of the Vagina.*—A woman, 25 years of age, who laboured under some slight disorder occasioned by errors of diet, took an emetic on the fourth day, which produced copious vomiting, and relieved her greatly.

A few days afterwards she began to complain of burning pain during micturition, and had some discharge of blood from the vagina, with severe pain in the external organs of generation. On examination, the external parts were found in a state of gangrene; on the following day the patient complained of an unusual feeling about the parts, and it was found that the whole vagina was prolapsed; as it was impossible to return it, antiseptic remedies were merely applied. The exposed parts now sloughed, and were finally completely removed on division of a band which retained them superiorly. The fever and other unfavourable symptoms quickly subsided; to prevent adhesion of the parts, a piece of sponge, moistened with some aromatic decoction, was introduced into the vagina. The vaginal portion of the uterus now became adherent to the upper edge of the vagina, while the lower remained free, and a new canal was formed, being merely somewhat shorter than the original vagina. The woman recovered perfectly, and the functions of the uterus were soon restored.—*Provin. Med. Jour.* July 2, 1842. *From Orvosi Tár, or the Hungarian Magazine of Health.*

